# Pharmaceutical Applications of Biomass Polymers: Review of Current Research and Perspectives

**DOI:** 10.3390/polym16091182

**Published:** 2024-04-23

**Authors:** Cornelia Bejenaru, Antonia Radu, Adina-Elena Segneanu, Andrei Biţă, Maria Viorica Ciocîlteu, George Dan Mogoşanu, Ionela Amalia Bradu, Titus Vlase, Gabriela Vlase, Ludovic Everard Bejenaru

**Affiliations:** 1 Department of Pharmaceutical Botany, Faculty of Pharmacy, University of Medicine and Pharmacy of Craiova, 2 Petru Rareş Street, 200349 Craiova, Dolj, Romania; cornelia.bejenaru@umfcv.ro (C.B.); antonia.radu@umfcv.ro (A.R.); 2 Institute for Advanced Environmental Research, West University of Timişoara (ICAM–WUT), 4 Oituz Street, 300086 Timişoara, Timiş, Romania; ionela.bradu@e-uvt.ro (I.A.B.); titus.vlase@e-uvt.ro (T.V.); gabriela.vlase@e-uvt.ro (G.V.); 3 Department of Pharmacognosy & Phytotherapy, Faculty of Pharmacy, University of Medicine and Pharmacy of Craiova, 2 Petru Rareş Street, 200349 Craiova, Dolj, Romania; andreibita@gmail.com (A.B.); george.mogosanu@umfcv.ro (G.D.M.); ludovic.bejenaru@umfcv.ro (L.E.B.); 4 Department of Analytical Chemistry, Faculty of Pharmacy, University of Medicine and Pharmacy of Craiova, 2 Petru Rareş Street, 200349 Craiova, Dolj, Romania; maria.ciocilteu@umfcv.ro; 5 Research Center for Thermal Analyzes in Environmental Problems, West University of Timişoara, 16 Johann Heinrich Pestalozzi Street, 300115 Timişoara, Timiş, Romania

**Keywords:** biomass, polymers, biodegradability, biocompatibility, pharmaceutical applications

## Abstract

Polymers derived from natural biomass have emerged as a valuable resource in the field of biomedicine due to their versatility. Polysaccharides, peptides, proteins, and lignin have demonstrated promising results in various applications, including drug delivery design. However, several challenges need to be addressed to realize the full potential of these polymers. The current paper provides a comprehensive overview of the latest research and perspectives in this area, with a particular focus on developing effective methods and efficient drug delivery systems. This review aims to offer insights into the opportunities and challenges associated with the use of natural polymers in biomedicine and to provide a roadmap for future research in this field.

## 1. Introduction

The shift toward environmental sustainability has catalyzed a marked transition to-ward natural biopolymers, moving away from the prevalent use of synthetic polymers across various sectors, including the pharmaceutical industry. Unlike their synthetic counterparts, biopolymers—synthesized through microbial, chemical, or natural processes—offer a promising alternative due to their comparable performance, versatility, and the potential for enhanced functionalities. These functionalities make biopolymers a critical asset in pharmaceutical, environmental, and medical applications, promising to significantly mitigate the issue of plastic pollution. Despite their vast potential, the adoption of biopolymers faces challenges, including high costs and inefficiencies in synthesis and processing, which must be overcome to realize their full potential [1,2,3,4].

Biomaterials, designed for direct interaction with biological systems, are at the forefront of medical innovation, enabling groundbreaking medical interventions through bio-compatible materials capable of performing specific functions. The success of these materials, especially in tissue engineering (TE) and drug delivery systems (DDSs), is intricately linked to their physical, chemical, and biological properties, necessitating meticulous customization to elicit desired responses from host systems. The use of polymers, characterized by their diverse and degradable properties, facilitates the creation of biomaterials that can disintegrate into low-molecular-weight products, either to be re-absorbed or excreted by the body, thereby enhancing their applicability and safety [5,6,7,8].

Natural polymers, categorized into proteins, polysaccharides, polyesters, lipids, or lignins (complex aromatic polymers), depending on their chemical structures, are utilized for biomedical purposes through copolymerization, merging polysaccharides like chitosan, starch, and cellulose with other polymers, and employing average protein-based biopolymers such as gelatin, collagen, and albumin for the creation of drug delivery nanomolecules due to their advantageous properties, such as minimal toxicity, narrowness, biodegradability, and prolonged stability [9,10,11].

Furthermore, biomass polymers’ inherent biocompatibility and functional chemical structures are used to develop nanomaterials for a broad range of biomedical applications, ensuring their efficient clearance from the body and eliminating the need for surgical retrieval. Degradable polymers undergo hydrolytic cleavage (enzymatic or nonenzymatic), producing soluble degradation products and enhanced properties like bioavailability, stability, and controlled release for applications in TE (e.g., cartilage scaffolds) and prosthetic implants. Refinement of the physical, chemical, and biological attributes of these polymers is achieved through strategies like blending, cross-linking, and forming interpenetrating polymer networks (IPNs), while recent efforts focus on synthesizing macromolecular biomaterials with optimized thermal and mechanical properties using chemical modification on their nanoconstructs. Innovative techniques, including physicochemical cross-linking, polyion complexes (PICs), layer-by-layer assembly, and nanoparticle (NP) coatings, have paved the way for developing sophisticated multiphase polymer systems tailored for specific biomedical uses [12,13,14,15].

Biopolymer composites enhanced with metals, natural fibers, and metal oxides represent a cutting-edge area of research, offering improved adsorptive, mechanical, and thermal properties. These advancements are supported by an array of characterization techniques, underscoring the ongoing efforts to develop cost-effective and performance-optimized biocomposites [16,17,18,19,20].

**Figure 1:**
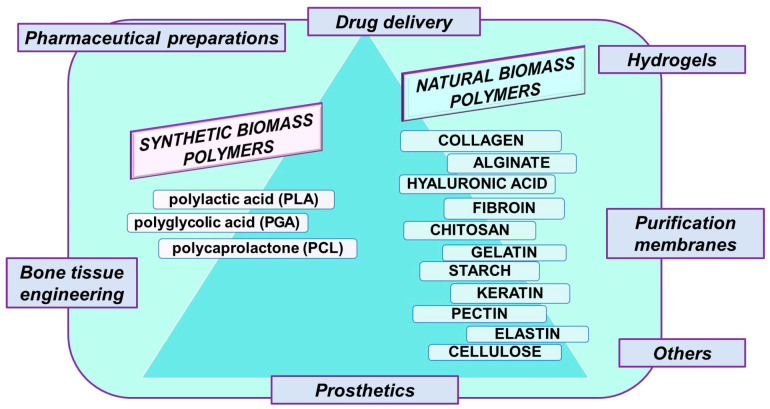
Schematic representation of principal biomass polymers and some of their main applications.

Natural biopolymers, like cellulose, starch, chitosan, and pectin, and synthetic biomass polymers such as polylactic acid (PLA), polycaprolactone (PCL), and polyglycolic acid (PGA), play crucial roles in various applications, including pharmaceutical preparations, purification membranes, hydrogels, prosthetics, drug delivery, and bone tissue engineering (BTE) (Figure 1).

These biopolymers demonstrate unique properties, with most of them being biodegradable, biocompatible, bioactive, or safe, filling the gap left by synthetic polymers. Enhancing the shelf life of food products, edible biopolymer packaging films derived from agricultural waste show nutraceutical, antimicrobial, and antioxidant properties. Furthermore, biopolymer particles generate gel-like structures in emulsion-based products, increasing texture, consistency, and stability [21,22,23,24].

## 2. Biopolymers

Biopolymers, originating from living organisms and composed of monomeric units such as amino acids, saccharides, and nucleic acids, are gaining prominence in medical and pharmaceutical industries, due to their key attributes: eco-friendliness, sustainability, biodegradability, non-toxicity, renewability, and compatibility. Biopolymers derived from natural biomass, exhibiting inherent biodegradability, are directly obtained from the natural environment and possess considerable economic value. Commercially, a myriad of biopolymers serves diverse purposes in biomedical devices, hygiene items, agriculture, and the food industry. Despite their advantages, the fabrication of biopolymers faces a major drawback in substantial financial expenditures, prompting ongoing research into cost-minimization methods, involving purification procedures, high-yielding microorganisms, and substrate selection [1,25,26].

In our study, we specifically focused on biopolymers extracted from biomass, sourced directly from food waste, organic, and other bio-based industries, emphasizing the use of polysaccharides, proteins, and lignin. These biopolymers, derived from natural and renewable sources, hold intrinsic properties that are pivotal for numerous applications within the pharmaceutical sector. Polysaccharides, with their diverse structural features and functionalities; proteins, known for their enzymatic activity and binding capabilities; and lignin, with its complex aromatic structure, offer a broad spectrum of applications ranging from DDSs to TE [27]. The choice of these biopolymers underscores our commitment to exploring sustainable and biocompatible materials that align with the principles of green chemistry and environmental stewardship, while harnessing their natural efficacy for innovative pharmaceutical applications.

Biopolymers are considered economic resources from diverse natural origins including plants, algae, microorganisms, animals, and agricultural residues. Green wastes can come from agricultural plant sources (cotton, tapioca, maize, bananas, cassava, potatoes, wheat, rice, and maize) or wood residues. In contrast, animal biopolymers mainly come from mammals (cattle, pigs) or marine sources (shrimps, lobsters, fish, sponges) [28,29,30,31]. Microbial origins (like fungi, algae, and yeasts) and vegetable oils extracted from meadow foam, fish, castor bean, linseed, tung, jojoba, rapeseed, safflower, sunflower, corn, and soybean may serve as rich sources of possible monomers, or co-monomers [32,33,34].

Biopolymers are categorized based on their biodegradability, origin, thermal response, and composition. The biodegradable and non-biodegradable distinction is evident, along with their classification as derived from natural origins or fossil fuels. The thermal condition response classifies them into thermosets, thermoplastics, and elastomers, while their composition leads to groups such as composites, laminates, and blends. Among the prominent systematization standards is the provenance of raw materials, resulting in natural, synthetic, and microbial biopolymers [1,35,36,37,38].

**Figure 2:**
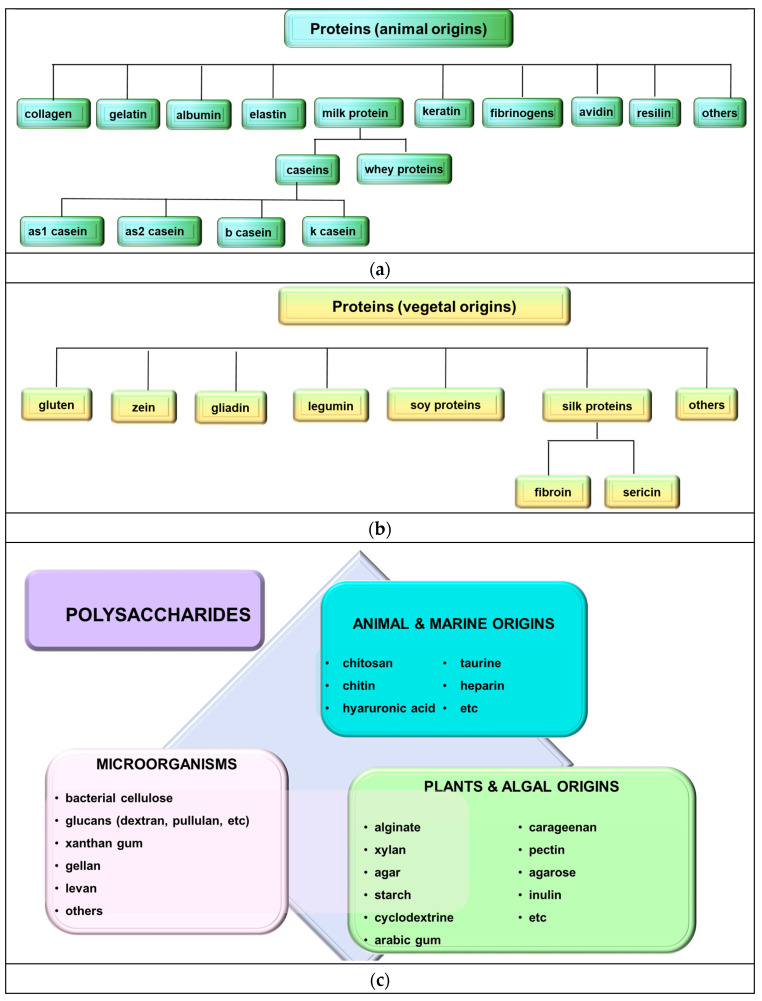
Naturally biomass polymers: proteins (**a**,**b**) and polysaccharides (**c**).

Naturally sourced biomass polymers (Figure 2a–c) encompass proteins (e.g., collagen, soy protein) and polysaccharides (e.g., chitosan, cellulose). Chemically synthesized polymers comprise PLA and petroleum-based polymers, such as polyethylene, PCL, and polyglutamic acid. Biopolymers from microbial generation, like polyhydroxyalkanoates (PHAs), bacterial cellulose, and gellan, serve diverse applications in medical, agro-industrial, and environmental sectors [1,35,36,37,38].

In the following sections, starting from recent studies, we selectively characterized various examples of the most relevant biopolymers used in the pharmaceutical domain.

### 2.1. Polysaccharides

Polysaccharides, natural polymers with functional hydroxyl, amino, and carboxylic groups, have garnered significant attention due to their inert, biocompatible, non-toxic, and cost-effective nature, coupled with excellent water stability. These versatile entities can be easily cross-linked, derivatized, or transformed into multiphase polymer systems such as polyblends, IPNs, graft, and block copolymers, while charged polysaccharides contribute to the formation of valuable PICs [39,40]. This discussion focuses on the key biomedical polysaccharides.

#### 2.1.1. Homoglycans

##### Starch

**Figure 3:**
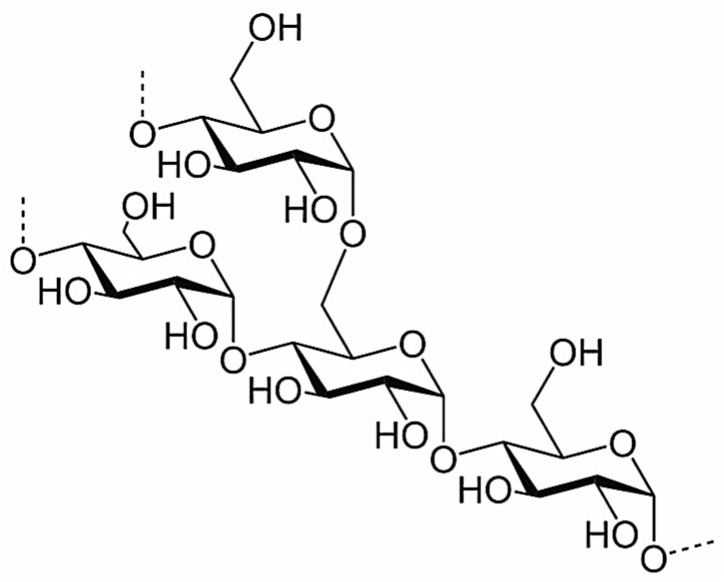
Starch—chemical structure.

Starch is a natural polysaccharide that comprises linear chains of amylose and branched amylopectin segments, primarily derived from α-glucose units and prominently present in cereal grains, fruits, roots, and legumes. Amylose features glucose units linked by α-(1,4) bonds, while amylopectin includes α-(1,4) and α-(1,6) linkages (Figure 3).

Properties of starch vary based on the plant source and its degree of maturity. Chemically altered starch, along with its physical mixtures or IPNs, serve as significant biomaterials in BTE, exhibiting enhanced characteristics. Nevertheless, starch’s inherent brittleness, premature degradation before reaching its melting temperature, inferior mechanical features, and challenging processability constrain its application as a standalone material [41,42,43,44].

##### Dextran

**Figure 4:**
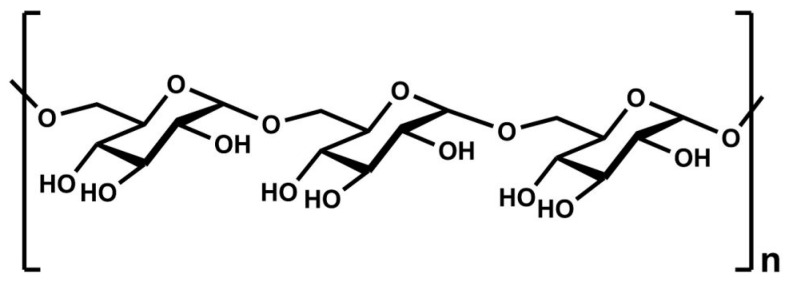
Dextran—chemical structure.

This bacterial homopolysaccharide encompasses glucans formed through the polymerization of α-D-glucopyranosyl moieties of sucrose catalyzed by dextransucrase enzyme. The main chain features glucose segments connected by α-(1,6) bonds, while branches exhibit α-(1,4), α-(1,3), and α-(1,2) units (Figure 4).

Properties like branching degree, molecular weight, and other attributes vary with the engaged microorganism. Dextran, with its remarkable rheological nature and plasma-volume-enlarging potency, undergoes chemical alterations, introducing thiol, (meth)acrylate, aldehyde, and phenol groups. As a biocompatible polysaccharide with no toxicity, dextran finds extensive applications in pharmaceutical and biomedical domains as an antithrombotic and bio-adhesive agent, in protein/drug delivery or tissue-engineered scaffolds. Injectable hydrogels are proposed as site-specific, trackable chemotherapeutic devices. Despite these advantages, challenges include high costs and limited availability [45,46,47,48].

##### Cyclodextrins

**Figure 5:**
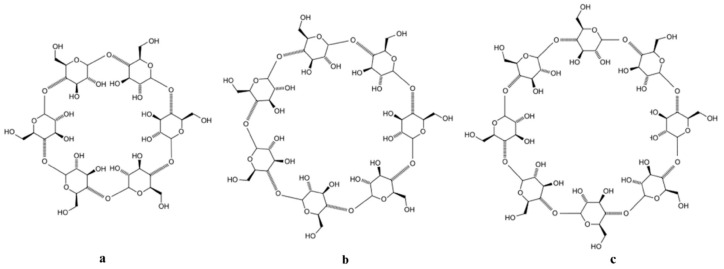
Chemical structure of α-cyclodextrin (**a**), β-cyclodextrin (**b**) and γ-cyclodextrin (**c**).

Cyclodextrins are oligosaccharides formed by the enzymatic linkage of glucose units (α-D-glucopyranose) through α-(1,4) bonds, resulting in the production of α-cyclodextrin (six units), β-cyclodextrin (seven units), and γ-cyclodextrin (eight units) (Figure 5a–c).

Characterized by a distinctive truncated cone-like structure, cyclodextrins feature an internal non-polar cavity with polar hydroxyl groups on the surface. This configuration allows hydrophobic substances, including drugs, to be encapsulated through hydrophobic interactions, forming host–guest supramolecular complexes driven by van der Waals and dipole–dipole interactions. The chemistry and applications of cyclodextrins have been subjected to comprehensive scrutiny across various fields. These inclusion complexes are amenable to derivatization and appropriate chemical alterations [49,50].

##### Cellulose

**Figure 6:**
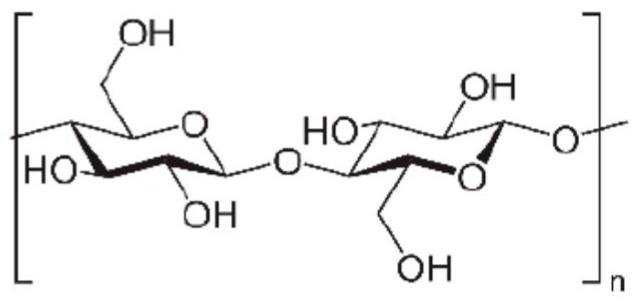
Cellulose—chemical structure.

The most abundant natural polysaccharide, cellulose, consists of β-D-glucopyranose units linked by β-(1,4) glycosidic bonds (Figure 6), offering significant potential as an advanced polymeric material.

Cellulose and its derivatives are versatile precursors; cellulose is generally well tolerated by the human body and other living organisms (in particular, cellulose ethers or esters), has low toxicity, and is a cost-effective material. Ionic liquids (ILs) and deep eutectic solvents overcome this issue. The derivatization of cellulose produces environmentally friendly materials, such as methylcellulose, cellulose acetate, hydroxypropyl cellulose, cellulose nitrate, and carboxymethylcellulose. Cellulose nanomaterials, including nanocrystals, bacterial nanocellulose (BNC), and nanofibrils, have been extensively researched. Nevertheless, cellulose has limitations such as low crease resistance, potential antigenicity, and lack of thermoplasticity [51,52,53].

##### Chitin and Chitosan

**Figure 7:**
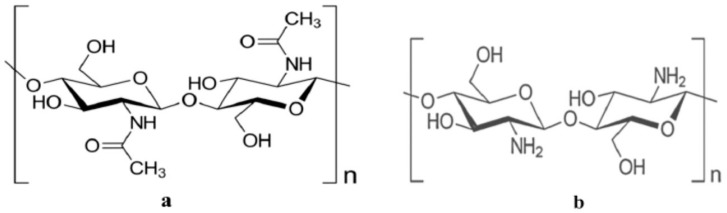
Chemical structure of chitin (**a**) and chitosan (**b**).

Chitin, a prominent constituent of sea crustacean shells, stands as the second-most abundant biomacromolecule utilized across various industries such as pharmaceuticals, textiles, food, and agriculture. Exhibiting biocompatibility, non-toxicity, biodegradability, and mucoadhesive properties, chitin can be effortlessly extracted and chemically altered to yield diverse biomaterials (Figure 7a).

Chitosan, extracted from crabs and fungal cell walls, undergoes commercial production through the deacetylation of chitin. The degree of deacetylation, impurity composition, and molar mass distribution are based on the natural source and preparation method. As a cationic linear copolymer polysaccharide, chitosan is composed of β-(1→4) connected 2-amino-2-deoxy-D-glucose (D-glucosamine) and 2-acetamido-2-deoxy-D-glucose (N-acetyl-D-glucosamine) segments through glycosidic bonds (Figure 7b). The polymer’s primary amino groups confer a positive charge on its surface, promoting inter- and intramolecular hydrogen bonding. Additionally, chitosan exhibits antimicrobial activity against viruses, fungi, and bacteria, rendering it valuable in the biomedical domain. However, its drawback lies in reduced solubility at physiological pH. Despite the variability in synthesis procedures, short-term human testing has shown no signs of allergic reactions [54,55,56,57,58].

#### 2.1.2. Heteroglycans

##### Alginate

**Figure 8:**
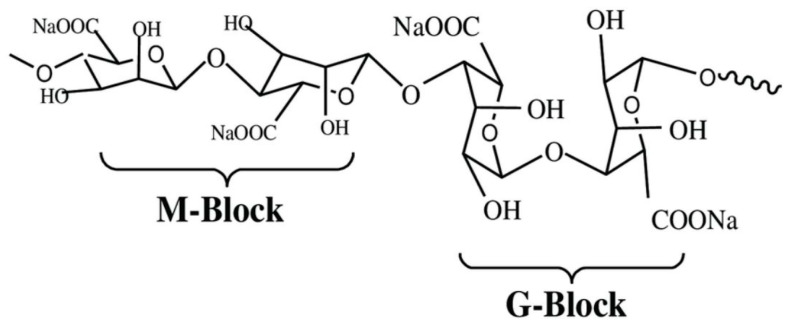
Sodium alginate—chemical structure.

Alginate, a water-soluble anionic polymer comprising α-L-guluronic acid (G) and β-D-mannuronic acid (M) residues connected by 1,4-glycosidic bonds, is biodegradable, biocompatible, and exhibits no toxicity (Figure 8).

Derived economically from marine brown algae, alginate finds diverse biomedical applications, serving as three-dimensional (3D) scaffolding materials in forms such as foams, microcapsules, sponges, and hydrogels for TE. Physical or chemical alteration enhances alginate’s properties, allowing the precise tuning of cell affinity, mechanical strength, and gelation through combinations with other biomaterials, ligand immobilization, and cross-linking. Despite its sensitivity to hydrolysis in acidic environments and challenges in fabrication due to reduced solubility, alginate-based materials have undergone clinical investigations, demonstrating potential benefits such as managing hypertension and advancements in the food industry [59,60,61,62].

##### Agarose

**Figure 9:**
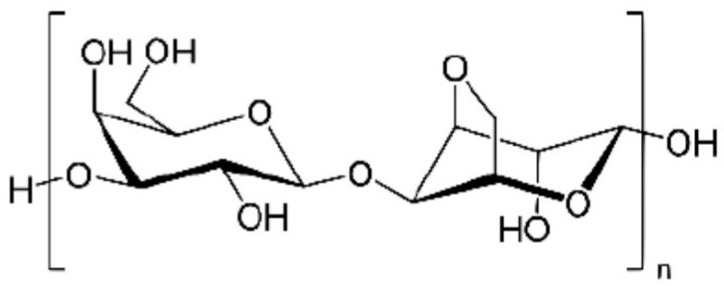
Agarose—chemical structure.

Agarose, an uncharged polysaccharide derived primarily from certain marine red algae, is a key component of agar. It is soluble in hot water, ILs, and polar non-aqueous solvents. From a structural standpoint, agarose is a linear and neutral polysaccharide comprising alternating (1,3)-β-D-galactopyranose and (1,4)-linked 3,6-anhydro-α-L-galactopyranose units (Figure 9).

Its solution forms gels upon cooling below ~40 °C, with flexible fiber chains capable of curling into helix structures, creating powerful gels with prominent hysteresis. Being non-toxic and biocompatible, agarose is commonly employed as a gelling agent in various applications, including chromatography techniques, nucleic acid electrophoresis, cell culture media, tissue culture overlays, and gel plates. Its exceptional properties, including mechanical resilience and reduced gelling temperature, make it suitable for applications like bio-ink, where gelation forms a 3D network of agarose fibers, disintegrating above 85 °C [63,64,65,66].

##### Carrageenans

**Figure 10:**
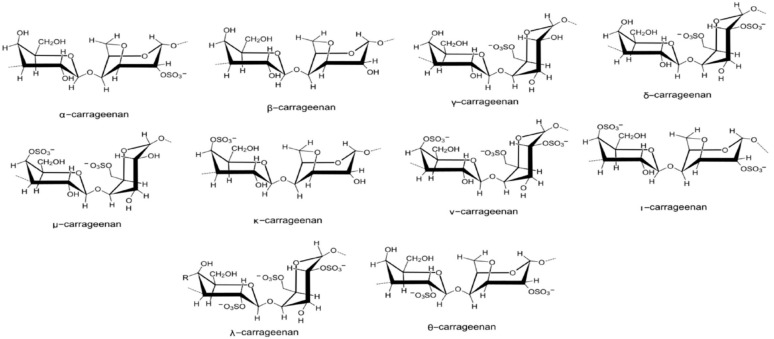
Chemical structures of main carrageenans.

Carrageenans, a family of linear sulfated polysaccharides extracted from red algae (*Rhodophyta*), known as Irish moss, exhibit extensive and very flexible molecules capable of forming helical structures, resulting in viscous solutions or elastic gels. Comprising alternate segments of β-D-galactose and 3,6-anhydro-α-D-galactose connected by α-(1,3) and β-(1,4) glycosidic bonds, carrageenans yield three main types—kappa (κ-1 sulfate group/disaccharide), iota (ι-2 sulfate groups/disaccharide), and lambda (λ-3 sulfate groups/disaccharide)—depending on the extraction method and algae source (Figure 10).

Beyond their applications in pharmaceutical, cosmetic, and food industries for colloid stabilization, thickening, protein binding, and gelling, carrageenans also influence plant growth stimulation and serve as pathogen resistance generators, offering crop protection. Despite their versatile properties, their reduced gel strength and anticoagulant effect remain as notable disadvantages [67,68,69,70].

##### Pectins

**Figure 11:**
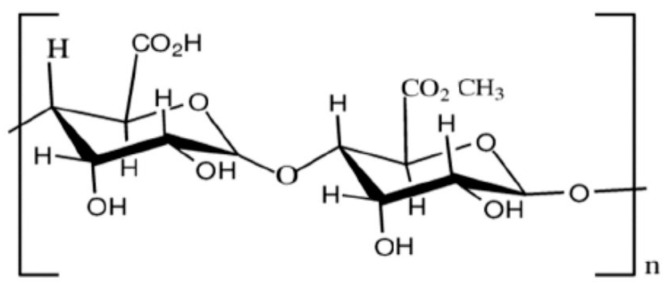
Chemical structure of pectins.

Pectins, polysaccharides found in the cell walls of superior plants, feature structures composed of D-galacturonic acid segments linked by α-(1,4) bonds, creating a linear chain framework with interrupted extensively branched regions. Variations in composition depend on the botanical origin (Figure 11).

Notably, pectins exhibit limitations such as a reduced water-vapor barrier and poor mechanical characteristics [71].

##### Arabic Gum

**Figure 12:**
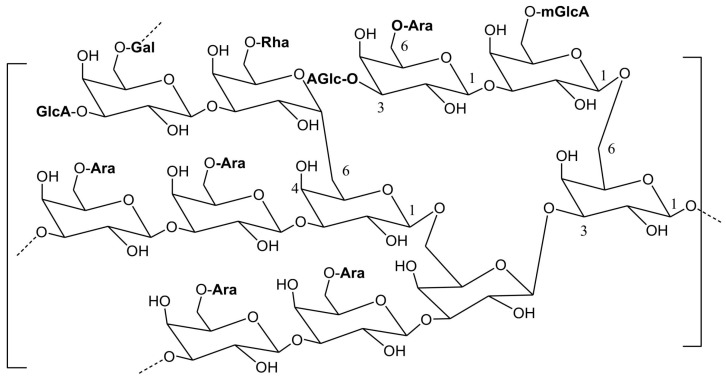
Chemical structure of Arabic gum.

Comprising a complex combination of glycoproteins and polysaccharides (Figure 12), prominently featuring arabinose and galactose, Arabic gum is a water-soluble neutral polymer widely employed as a thickener, stabilizer, and emulsifier in the pharmaceutical, cosmetics, and food industries.

Beyond its conventional uses, Arabic gum serves as a versatile excipient, contributing to the development of nanoscale scaffolds for drug delivery and biomedical practice. Strategies include cross-linking to form hydrogels, combining with other polymers, creating drug conjugates, and attaching to NPs, showcasing its potential biomedical implementation [72,73,74,75].

##### Guar Gum

**Figure 13:**
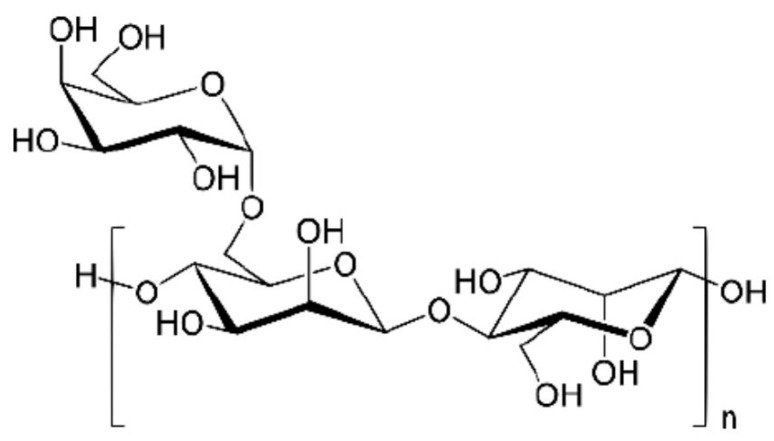
Chemical structure of guar gum.

Guar gum is a water-soluble polysaccharide with a high molecular weight, extracted from the seeds of *Cyamopsis tetragonolobus*. It consists of a primary chain of D-mannopyranose residues linked by β-(1,4) glycosidic bonds, connected to D-galactopyranose residues through α-(1,6) glycosidic bonds (Figure 13).

Known for its emulsifying, thickening, and stabilizing properties, guar gum finds applications in the food, pharmaceutical, and cosmetic industries. Its cold-water solubility is influenced by the galactose/mannose molar ratio. Modified through functionalization (carboxymethylation, hydroxyalkylation, or esterification), guar gum is tailored for biomedical applications, enhancing its mechanical features and reducing aqueous solubility. Guar gum and its derivatives are particularly suitable for oral drug delivery due to their heightened stability across a large pH range [76,77,78,79].

##### Inulin

**Figure 14:**
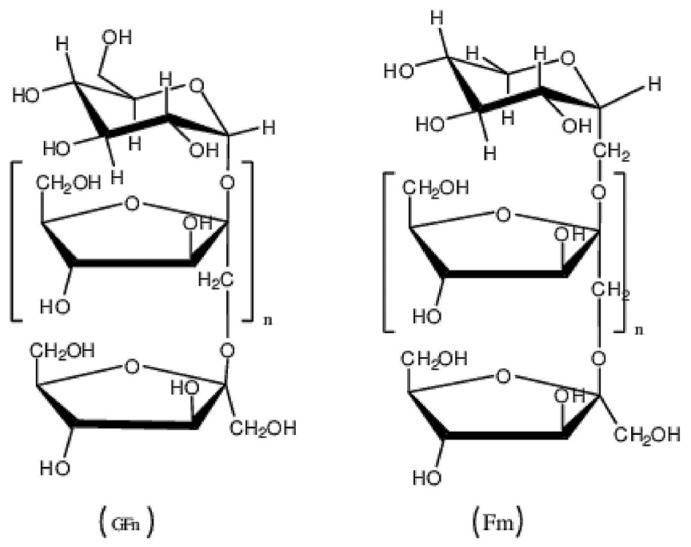
Chemical structure of inulin.

Inulin is a natural, inexpensive polysaccharide composed of fructose chains joined by β-(2-1) bonds with a glucose terminal unit (Figure 14).

The applications of this biopolymer are multiple, especially in prebiotics and nutraceuticals. The latest research reported various potential biomedical applications: the development of target delivery systems (stable against the action under low pH and the action of specific enzymes such as pepsin and lipase) for colon cancer strategies, nanocarriers with antitumor and antioxidant activities, increasing calcium absorption, and others [80].

##### Glycosaminoglycans

Hyaluronic Acid

**Figure 15:**
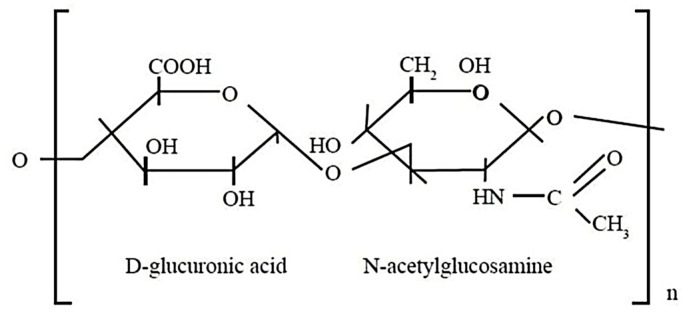
Chemical structure of hyaluronic acid.

Hyaluronan or hyaluronic acid (HA) is a non-sulfated glycosaminoglycan with a linear structure, consisting of disaccharide repeat segments of β-1,4-D-glucuronic acid and β-1,3-N-acetyl-D-glucosamine connected by β-1,4-glycosidic bonds (Figure 15).

Predominantly found in the extracellular matrix of vertebrate soft connective tissues, HA plays a crucial role in tissues like the umbilical cord, synovial fluid, skin, and vitreous humor. Commercially sourced from rooster combs or bacterial fermentation, it is an anionic polysaccharide with the ability to absorb a significant amount of water, serving as a lubricant in native extracellular matrixes and influencing connective tissue viscoelasticity. HA has the potential for chemical alteration through processes such as cross-linking and grafting. In numerous tumor and inflammation conditions, cluster of differentiation (CD)44 and CD168 serve as major ligands for HA. It also plays pivotal roles in biological processes such as cell proliferation, tumor invasion, tissue homeostasis, angiogenesis, and matrix organization through its interactions with cells. Despite its susceptibility to accelerated degradation, HA is extensively used in applications such as drug delivery, TE, and cutaneous rejuvenation. Challenges lie in the brittleness and aqueous solubility of HA hydrogels, leading to the development of useful biomaterials like derivatized HA and IPNs/PICs of HA, albeit facing issues of increased cost and inferior mechanical features [81,82,83,84,85].

Chondroitin

**Figure 16:**
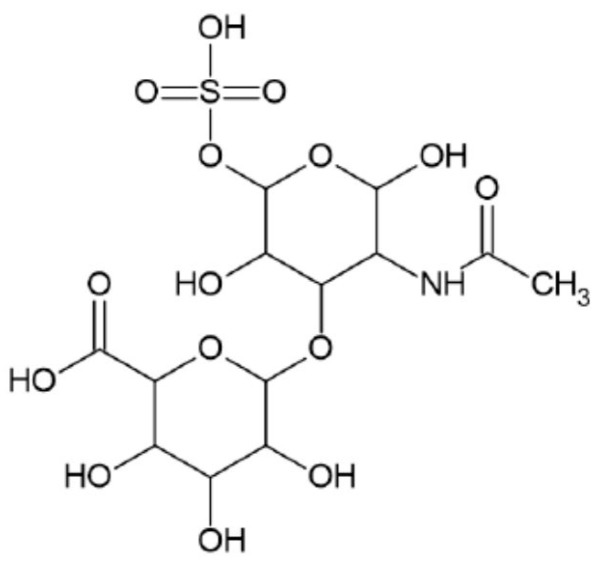
Chemical structure of chondroitin sulfate.

Chondroitin sulfate, a primary component of hyaline cartilage in cartilage and at the bone calcification location, is a sulfated glycosaminoglycan with recurrent disaccharide segments of β-1,4-linked-D-glucuronic acid and β-1,3-linked N-acetyl galactosamine, featuring certain sulfated positions (Figure 16). The two major chondroitin sulfates vary in sulfate positions at 4 or 6.

With polar carboxyl and hydroxyl groups, the polymer exhibits covalent/electrostatic interactions with other materials. Possessing antithrombosis, negative immunogenic, anticoagulant, antioxidant, and antiatherosclerosis actions, chondroitin sulfate serves as a valuable biomaterial. Widely employed in osteoarthritis treatment, it can target CD44 receptors on tumor cells, making it applicable for cancer management [86,87,88,89,90,91].

### 2.2. Proteins

#### 2.2.1. Collagen

**Figure 17:**
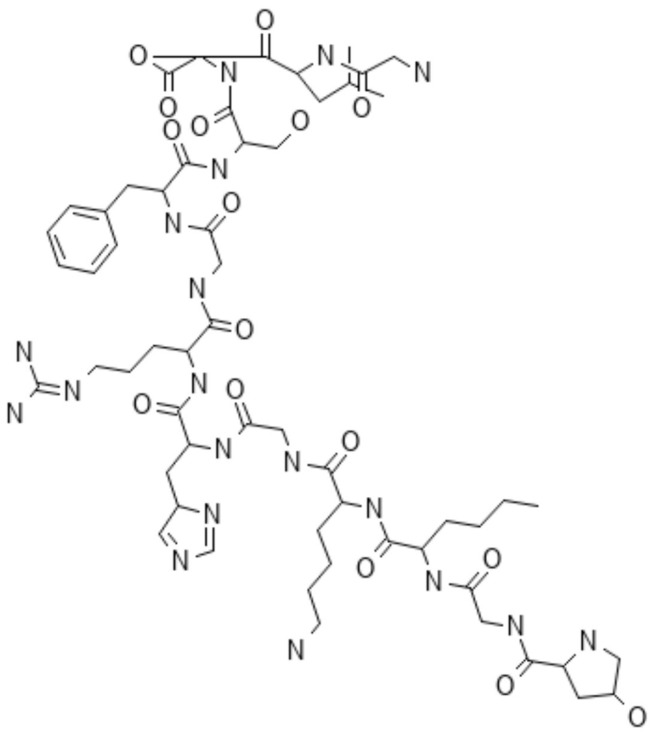
Chemical structure of collagen I (α chain).

Collagen is the oldest protein structure identified in dinosaur fossils. Approximately 30% of the total animal protein is represented by collagen, which is indispensable in maintaining the biological integrity of the connective tissues. Currently, there are 29 types of collagens, characteristic of different tissues of the human body. Figure 17 shows the chemical structure of collagen I (α chain).

The predominant sources of collagen, mainly from bovine origin due to favorable biocompatibility and low immunogenicity, with potential alternatives from marine organisms, are commonly utilized despite associated challenges such as difficulty in sterilization, susceptibility to bacterial contamination, batch variability, and immunogenicity, necessitating ongoing research into the extraction, purification, and industrial-scale production of modified recombinant collagen [92,93]. Common collagen extraction methods involve solubilization in neutral saline, acidic solutions, and acidic solutions with enzymes, albeit at high costs due to requisite chemical treatments for bond elimination, crucial for yield optimization in research-oriented collagen production. Marine collagen, while offering biological safety without disease transmission risks, exhibits lower stability attributed to a lower denaturation temperature compared to mammalian collagen [94,95].

Collagen’s amino acid composition, varying across species, influences its physical and chemical properties, thermal stability, solution viscosity, and cross-linking potential, enabling its utilization in wound healing, ophthalmic treatment, drug delivery, and genetic engineering. Key considerations for employing collagen in biomaterial matrices include thermal stability, mechanical resistance, and specific biomolecular interactions. Its excellent biocompatibility and biodegradability render it ideal for various medical implants, such as porous sponges, membranes, and surgical threads, as well as cell culture substrates. While collagen-based supports often incorporate synthetic components for enhanced mechanical strength, they serve diverse purposes, including drug delivery, TE, and epithelial barrier formation to promote tissue regeneration. Despite collagen’s inherent biological advantages, its mechanical properties and structural stability may require enhancement through cross-linking treatments, allowing for tailored matrix modifications without compromising cellular responses. Combining natural and synthetic polymers further expands the potential of collagen-based systems to address multifaceted biomedical needs [96,97,98,99,100,101].

Both naturally derived and recombinant forms of collagen hold significant value as biomaterials, widely utilized in diverse fields such as TE and cosmetic surgery. Recognized by regulatory authorities like the U.S. Food and Drug Administration (FDA), collagen’s versatility extends to its incorporation into composite materials with hydroxyapatite and tricalcium phosphate as a biodegradable synthetic bone graft alternative and its use in various drug and gene delivery applications. To conclude, collagen’s adaptable utility underscores its indispensable role in biomedical advancements and therapeutic interventions [102,103,104,105,106].

#### 2.2.2. Gelatin

**Figure 18:**
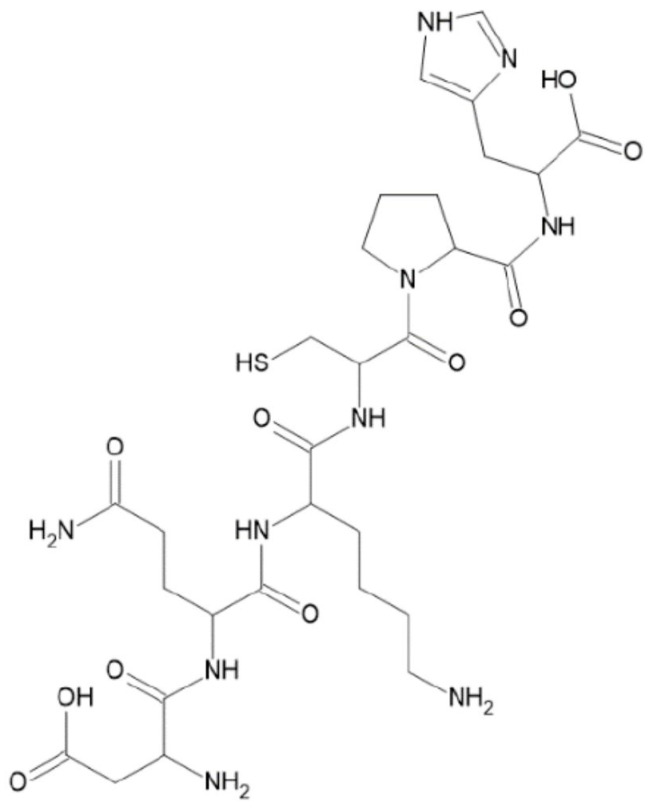
Chemical structure of gelatin.

Gelatin, a naturally occurring biopolymer derived from collagen, is abundant in connective tissues, skin, and bones, finding extensive utility across the food, pharmaceutical, and cosmetic industries. Resulting from the partial hydrolysis of collagen, gelatin comprises a heterogeneous ensemble of peptides and proteins, characterized by its hydrophilic nature due to the presence of numerous amino and hydroxyl groups, facilitating water absorption and gel formation (Figure 18).

Its biocompatibility, biodegradability, and capacity for forming stable hydrogels have positioned gelatin as a prominent candidate for DDSs, particularly due to its thermo-reversible properties, enabling gel formation at physiological temperatures suitable for injectable drug delivery applications. Furthermore, gelatin’s versatility allows for facile modification to achieve specific drug release profiles through chemical cross-linking or blending with other polymers, thus tailoring mechanical and release properties as needed for diverse therapeutic applications. Additionally, gelatin’s inherent bioactivity supports cell adhesion, rendering it suitable for TE endeavors, with the integration of bioactive molecules enhancing its potential for regenerative medicine and targeted drug delivery. Nevertheless, challenges such as potential immunogenicity and rapid in vivo degradation necessitate strategies such as cross-linking and polymer blending to address these limitations and ensure the stability and sustained release of encapsulated drugs [107,108,109,110,111].

Cross-linking serves as a pivotal mechanism in bolstering the stability of gelatin structures, averting premature degradation and upholding the integrity of DDSs. Through cross-linking, the porosity and mesh size of gelatin matrices are modulated, thereby influencing the diffusion kinetics of drugs and affording precise control over release mechanisms. This flexibility enables the creation of tailored release profiles, encompassing sustained, controlled, and stimuli-responsive release patterns. Researchers adeptly manipulate the hydrophilicity, mechanical strength, and degradation rate of gelatin matrices by judiciously selecting cross-linking agents and methodologies [112,113,114].

Blending gelatin with other polymers represents a prevalent strategy aimed at augmenting the properties and performance of gelatin-based materials, particularly within DDSs. The selection of polymers for blending hinges upon the desired characteristics of the resultant composite material. Notably, poly(lactic-*co*-glycolic acid) (PLGA) is frequently amalgamated with gelatin to bolster mechanical strength and regulate degradation rates. Conversely, polyethylene glycol (PEG), another hydrophilic polymer, serves to enhance the water solubility and stability of gelatin-based materials, concurrently mitigating protein adsorption, thereby offering potential benefits in specific biomedical applications. Furthermore, the blending of gelatin with chitosan finds extensive exploration in crafting wound dressings, TE scaffolds, and DDSs [115,116,117].

#### 2.2.3. Silk Protein

**Figure 19:**
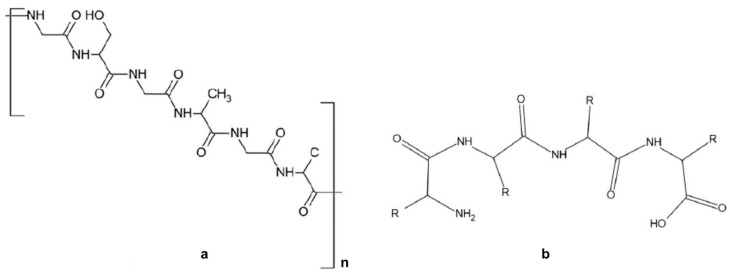
Chemical structure of fibroin (**a**) and sericin (**b**).

Insects, such as silkworms and spiders, generate silk, the most robust natural protein fiber, known for its exceptional mechanical features, including flexibility, increased tensile strength, biodegradability, resistance to compression, and reduced immunogenicity, making it biomedically significant. Silkworm silk consists mainly of fibroin (Figure 19a) and sericin (Figure 19b), with fibroin exhibiting histocompatibility, hydrophobicity, minimal immunogenicity, non-toxicity, and insolubility.

Derived from silkworm cocoons, fibroin, composed of amino acids (alanine, serine, and glycine), forms various structures like NPs, fibers, gels, hydrogels, scaffolds, and membranes. Its biodegradability and biocompatibility, along with its mechanical strength and malleability, position it as a promising candidate for the drug delivery domain. Sericin, a water-soluble hydrophilic protein, acts as a glue, offering intrinsic antioxidant and anticancer properties in its NP form [118,119,120,121,122].

#### 2.2.4. Albumin

**Figure 20:**
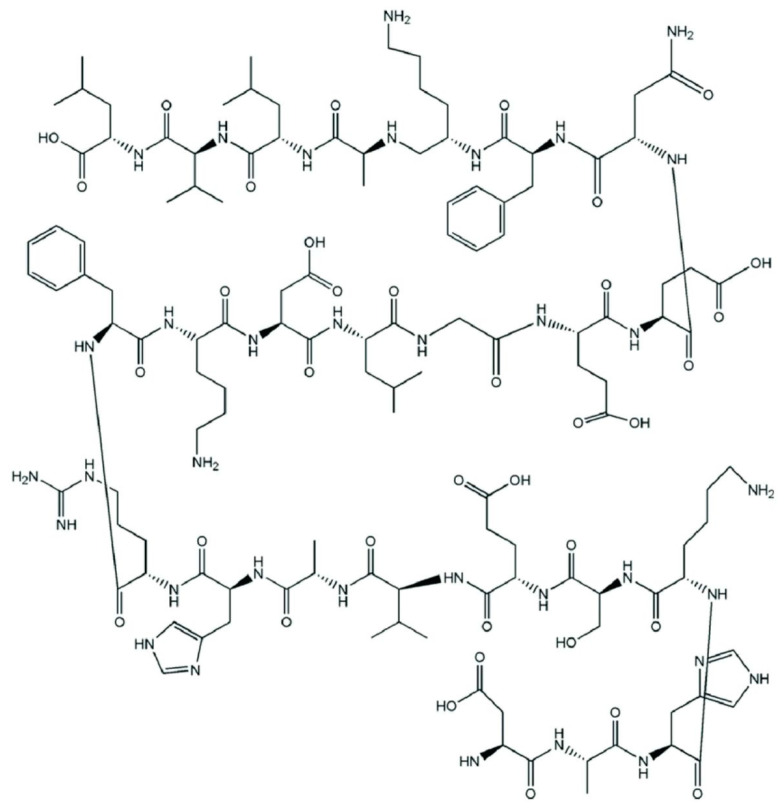
Chemical structure of albumin (serum albumin).

A protein existing in both animal and plant physiological fluids/tissues, albumin plays crucial roles such as maintaining osmotic pressure, neutralizing free radicals, and connecting and transporting numerous substances like drugs and hormones in the circulatory system. It acts as an interface between cells and scaffold materials like collagen, facilitating their integration in TE. Serum albumin, a biodegradable, stable, and non-toxic protein, significantly influences pharmacokinetics and drug distribution/metabolism through drug attachment. Consequently, albumins have surfaced as prospective drug carriers, finding implementation in biosensors, contrast agents, theranostics, and implants for various conditions. The structure and functional groups of albumins (Figure 20) enable the linking and capping of inorganic NPs, increasing compatibility and bioavailability, with low toxicity and selective bioaccumulation [123,124].

##### Bovine Serum Albumin

Bovine serum albumin (BSA) plays a pivotal role in fetal bovine serum (FBS) used in vaccine production, necessitating its accurate detection for compliance with regulatory standards. Molecularly imprinted polymers (MIPs), inspired by Fischer’s lock and key theory, have emerged as promising artificial receptors for protein detection, offering high selectivity and affinity. Their stability, simplicity in preparation, and adaptability for diverse applications, including vaccine production and clinical diagnostics, position MIPs as valuable tools in biomedical and laboratory settings, facilitating sensitive assays and detection methodologies [125,126,127,128].

Human serum albumin (HSA) and BSA are extensively studied major serum proteins, with HSA constituting about 60% of human blood serum, playing a multifunctional role in transporting various substances and influencing their solubility and distribution in the body through its heart-shaped globular structure containing three main domains and two binding sites, while BSA, sharing structural similarities with HSA, is characterized by its acidic nature and negatively charged hydrophobic cavities. Both albumins have applications in biological and medical fields, including the preparation of albumin NPs such as Abraxane for treating metastatic breast cancer [129,130,131,132,133].

Molecular imprinting technology (MIT), recognized for its utility in crafting selective artificial receptors for sensing target molecules, operates by creating specific cavities within a polymer matrix through a three-step process involving the arrangement of functional monomers around the template molecule, polymerization in the presence of cross-linker monomers, and template removal. MIT employs two main strategies, covalent and non-covalent imprinting, with covalent imprinting forming well-designed cavities but requiring harsh conditions for template removal, while non-covalent imprinting offers a milder approach but may exhibit potential reversibility in complex formation, prompting the introduction of semi-covalent imprinting, which combines the advantages of both methods, with an effective modification employing a “sacrificial spacer” to enhance precision in molecular imprinting [134,135]. Despite advancements in molecular imprinting, persistent challenges exist in developing artificial materials for detecting biological macromolecules like BSA, primarily due to their molecular instability, conformational flexibility, large size, diverse functional groups, and the need for mild imprinting conditions associated with proteins, which often require aqueous environments for stability, prompting the exploration of alternative techniques such as surface molecular imprinting technology (SMIT), epitope-mediated imprinting, micro-contact imprinting, imprinted ILs, and imprinted hydrogels to improve the efficiency of prepared MIPs for selective BSA detection [136].

SMIT has been employed to develop BSA-MIPs, offering benefits such as the homogeneous distribution of binding sites, improved mass transfer, and enhanced adsorption dependency by creating molecular recognition sites on the support substrate surface, notably making the created molecular recognition sites readily accessible to protein molecules [137,138,139,140,141].

ILs have enabled the development of sensitive electrochemical sensors for detecting BSA, with chitosan/IL–graphene-modified electrodes and MIPs showing promising results. Molecularly imprinted hydrogels, responsive to environmental stimuli, offer a dynamic platform for selective protein recognition, particularly with temperature-sensitive components. Utilizing sodium alginate and thermo-sensitive polymers, high-toughness hydrogel films were prepared, exhibiting enhanced BSA adsorption capabilities. Additionally, surface-imprinted materials incorporating hollow magnetite microspheres demonstrated specific BSA recognition, highlighting their potential for bioseparation and biosensor development [142,143,144,145].

##### Other Proteins

Zein

Zein is an amphiphilic protein group consisting of α, β, γ, and δ zein in various proportions, with a predominant proportion being α-zein (about 80%) followed by δ-zein. Zein represents about 50% of the whole protein content in corn (*Zea mays*). The unique properties of zein, such as its solubility, are due to its high proportion of hydrophobic, neutral amino acids like alanine, leucine, and proline, as well as the presence of polar amino acids such as glutamic acid (approximately 20% of its total amino acid content). Its unique physical properties, such as its high thermal and water stability and an isoelectric point (about 6.8) very close to the physiological pH value, are due to the presence of numerous and varied types of functional groups (amines, amides, hydroxyls, carboxylates, and phenols). Zein is used extensively in edible film preparation for the biomedical area and food industries. Recent studies reported the development of new nano- and micromaterials for target drug delivery, imaging, theranostics, and TE [146,147,148].

Legumin

**Figure 21:**
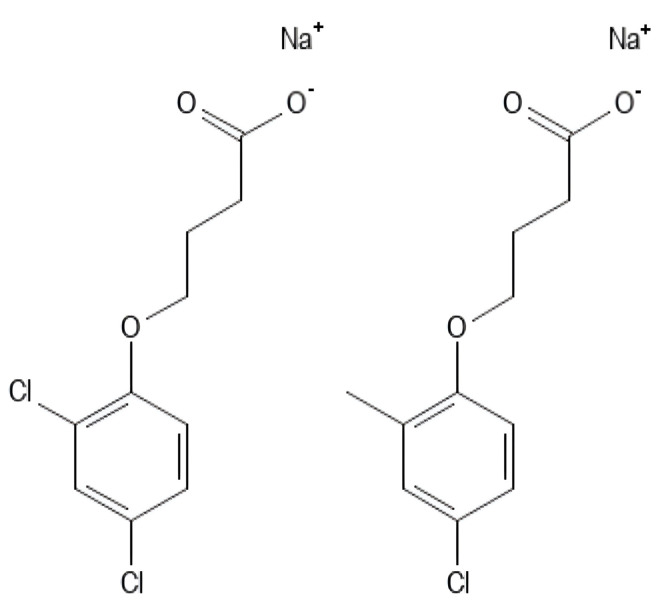
Chemical structure of legumin.

Legumin is a vegetable protein that contains numerous sulfur-amino acids, with a structure (Figure 21) similar to casein.

It is abundant in soybean seeds, beans, peas, lentils, vetches, and hemp. In recent years, several advanced materials based on legumin have been used in nutraceuticals and biomedical applications [149,150].

Gliadin

**Figure 22:**
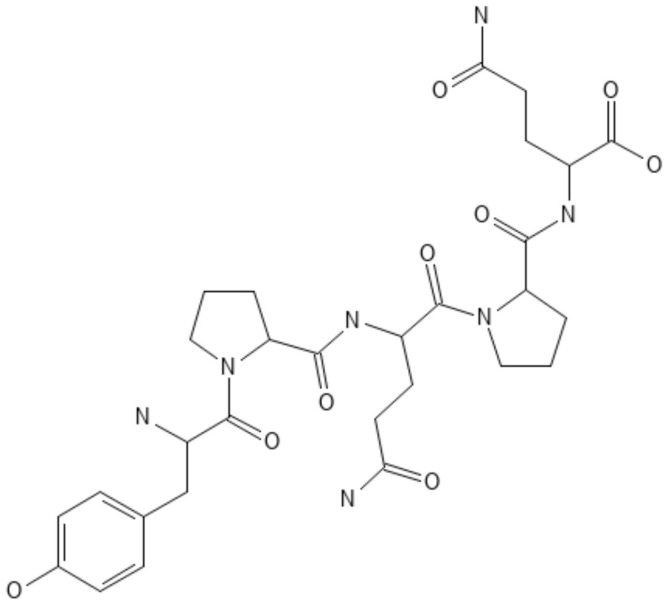
Chemical structure of gliadin.

Gliadin proteins (classified as α, β, γ, and ω gliadins) are found in wheat. Among the most alluring properties, from the point of view of the application potential in the biomedical field, are their low water solubility at ordinary pH values (because of its chemical structure (Figure 22) consisting of single-chain polypeptides linked by intramolecular disulfide bonds), high biocompatibility, non-toxicity, and biodegradability [151,152].

Recent studies have exploited these characteristics of gliadin for the development of new oral and local DDSs for gastrointestinal (GI) diseases, breast tumors, etc. [151,152,153].

Avidin

Avidin is a basic, homogeneous glycoprotein consisting of tetrameric biotin-binding protein and about 10% carbohydrate moieties (4–5 mannose and 3 N-acetylglucosamine residues) derived from egg whites.

Avidin is extremely water-soluble and shows high stability in a wide range of pHs and temperatures. Therefore, this biopolymer has gained multiple applications in bio-chemical assays, diagnosis, drug delivery, etc. In recent studies, it was employed for its versatile functionality to obtain new nanomaterials for nano-DDSs and diagnosis [154].

### 2.3. Lignin

**Figure 23:**
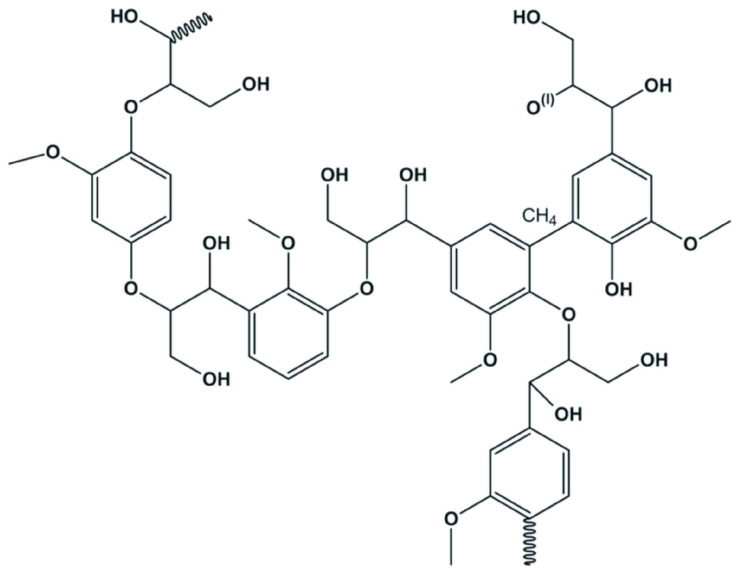
Chemical structure of lignin.

Lignin, comprising up to 35% of lignocellulosic biomass, is the second-most abundant biopolymer, after cellulose (Figure 23) [155,156,157,158].

Lignin is considered toxic due to its complex, recalcitrant, and xenobiotic nature, which makes it resistant to enzymatic and microbial degradation. This resistance stems from lignin’s complex aromatic structure, which is not readily broken down by most microorganisms. As a result, lignin and its derivatives can accumulate in the environment, posing a challenge for biological systems and microbial communities that are unable to process these compounds efficiently [159]. The main source of toxic lignin comes from lignocellulosic biomass, which is a complex and abundant group of organic materials composed primarily of cellulose, hemicellulose, and lignin. Lignocellulosic biomass is found in agricultural residues, forestry waste, certain grasses, and other plant materials. Among these components, lignin is particularly notable for its complex, amorphous, and recalcitrant nature, which makes it resistant to degradation by enzymes and microorganisms. The paper and pulp industries are significant contributors to lignin generation, as lignin is separated from cellulose during the process of paper production, leading to substantial amounts of lignin by-products. This lignin by-product is often considered waste, although it has the potential for conversion into valuable bioproducts through various biochemical and thermochemical processes. The study conducted by Mohammad and Bhukya (2022) [159] delves into this challenge, presenting a novel biotransformation approach that leverages the capabilities of *Pseudomonas putida* KT2440. This bacterium exhibits remarkable tolerance to high concentrations of lignin and its aromatic derivatives, converting these toxic compounds into eco-friendly biopolymers. The key to transforming lignin into biocompatible materials lies in the acclimatization process and the strategic addition of glucose, which significantly enhances the degradation capability of the strain. This breakthrough underscores a dual benefit: detoxifying the environmental menace posed by lignin and its derivatives while simultaneously synthesizing valuable biopolymers [159].

Lignin exhibits antioxidant, antibacterial, and anti-ultraviolet activities attributed to its unique polyphenolic structure. Nonetheless, the heterogeneity of lignin derived from varied sources and extraction methods poses a significant challenge to its use in the biomedical field [160,161].

In recent times, research studies have been focused on the chemical modification of lignin through techniques such as alkylation, esterification, phenolation, etherification, and urethanization. This approach enhances lignin’s solubility, thermal stability, and reactivity while reducing its brittleness, thereby enabling the development of advanced nanomaterials such as lignin microcapsules, self-assembling NPs, lignin-based complex micelles, lignin-based carbon dots, and biosensors for a wide range of applications, including drug delivery, gene delivery, biosensors, bioimaging, TE, and dietary supplements [162].

### 2.4. Shellac

Shellac is a composite macromolecule (a long-chain polyester type of resin) consisting of inter- and intra-esters of polyhydroxy carboxylic acids (aliphatic long-chain hydroxy acids and sesquiterpene acids). Unique features of this biopolymer, namely its thermoplasticity, non-toxicity, and water stability at neutral to acidic pHs, determined its use in the medicine and food industries. The latest research reported the development of various tailored DDSs based on shellac [163].

## 3. Chemical Modifications of Biopolymers

### 3.1. Cross-Linking

Cross-linking involves the formation of a network in polymer solutions, enhancing mechanical features and viscoelastic behavior. The unstable bonds, produced through physical or chemical cross-linking, can be disintegrated under physiological conditions. Chemical cross-linking, utilizing covalent agents, enhances mechanical stability but may impact polymer integrity and increase toxicity. The ionic gelation procedure involves interactions between polymers with opposite charges and cross-linking agents with complementary charges. In contrast, polyelectrolyte complexation relies only on electrostatic interactions among positively or negatively charged polyions, without the use of cross-linking agents. Innovative dual cross-linking combines both physical and chemical factors, reducing toxicity and improving stability. Interfacial cross-linking allows nanocapsule preparation without additional agents. Alginate readily cross-links via ionic interactions with calcium ions, forming gels employed for encapsulating bioactive molecules. Cationic polysaccharides like chitosan can be cross-linked with glycerol-phosphate disodium salt [164,165,166,167,168].

### 3.2. Functionalization and Conjugation

Biopolymer conjugation refers to the covalent attachment or linking of two or more biopolymers through specific chemical reactions or cross-linking mechanisms. The new resulting polymer structure has enhanced properties or functionalities. Biodegradable polymers, especially polysaccharides, possess diverse functional groups that can undergo covalent modifications with hydrophobic or hydrophilic substances, enhancing their suitability for biomedical approaches. Chemical conjugation, such as the PEGylation of polysaccharides or proteins (–OH groups of PEG react with –COOH, –NH_2_, or –SH groups on the target molecule), can modify the physical properties and solution behavior for specific utilization (Table 1). Various strategies of functionalization, including etherification, esterification, and enzymatic modifications, yield polysaccharide derivatives with improved biological, chemical, and physical features. Reactive functional groups introduced by phosphorylation, sulfation, acylation, and alkylation significantly impact inherent hallmarks. Additionally, enzymatic modifications, involving glycosylation, oxidation, and molecular weight depletion, have been designed for diverse pharmaceutical employments [169,170,171,172,173,174].

### 3.3. Interpenetrating Polymer Networks

IPNs consist of two or more incompatible polymers synthesized together, with one system polymerized in the presence of another. For instance, an aqueous solution containing a water-soluble polymer and a vinyl/acryl monomer can be polymerized to form intertwined polymer chains. Unlike polymer blends, IPNs expand but do not dissolve in solvents, reducing flow/creep conduct. They rely on physical forces like electrostatic and hydrogen bonding, making them suitable as vehicles for DDSs and scaffolds for TE. IPN hydrogels are innovative biomaterials for drug delivery, with polysaccharide-based IPNs, particularly using chitosan and alginates, offering a unique enlargement ability, mechanical strength, and specificity [175,176,177,178,179,180].

### 3.4. Graft Copolymers

Grafting serves as a versatile strategy to enhance the compatibility between synthetic and natural polymers, particularly in the chemical alteration of polysaccharides. Various polysaccharides, like cellulose, HA, chitosan, and starch, have been successfully employed in grafting processes. The “grafting through/on/from” approaches enable the incorporation of hydrophilic or hydrophobic polymeric moieties onto the polysaccharide backbone, with microwave irradiation emerging as an efficient method, offering improved attributes in terms of flame resistance, water repellence, thermal stability, and opposition to acid–base aggression. Polysaccharide-based graft copolymers, especially those with amphiphilic characteristics, find relevance in the biomedical domain, showcasing potential as biomaterials or conveyances for DDSs [181,182,183,184,185,186].

### 3.5. Block Copolymers

Biodegradable block copolymers have garnered significant attention in medical and pharmaceutical studies on account of their customizable biodegradability, biocompatibility, and self-assembly characteristics. These polymers serve as effective vehicles for DDSs, forming drug-loaded NPs that undergo degradation in biological circumstances and are subsequently evacuated via the renal system. The precise control over the structure of block copolymers, achieved through advancements in polymerization techniques like atom transfer radical polymerization (ATRP) and reversible addition–fragmentation chain-transfer (RAFT) processes, coupled with modern nanoaggregate interpretation methods, has heightened their relevance in various biomedical applications [187,188,189].

### 3.6. Polyion Complexes

Polyelectrolytes, water-soluble charged polymers, undergo dissociation in aqueous solutions, resulting in the formation of a macroion and counterion. The conduct of polyelectrolytes in solution is significantly affected by the existence of salts, as well as pH and temperature variations. Natural polyelectrolytes find extensive applications in various industries. Charged polymers interrelate with oppositely charged ones, forming soluble PICs or insoluble coacervates, which have biological significance. Examples include PICs of oppositely charged polysaccharides like chitosan and alginates, and those involving charged proteins/polysaccharides and oppositely charged small molecules or polymers. Dilute solutions of polyelectrolytes, when mixed with oppositely charged substances, can spontaneously form a new phase via powerful electrostatic interactions. Nevertheless, a comprehensive interpretation of the physical status (solid/liquid-like) is essential. The presence of supporting electrolytes significantly affects the origination and qualities of these complexes [187,190,191,192,193].

## 4. Pharmaceutical and Biomedical Applications of Biopolymers

**Figure 24:**
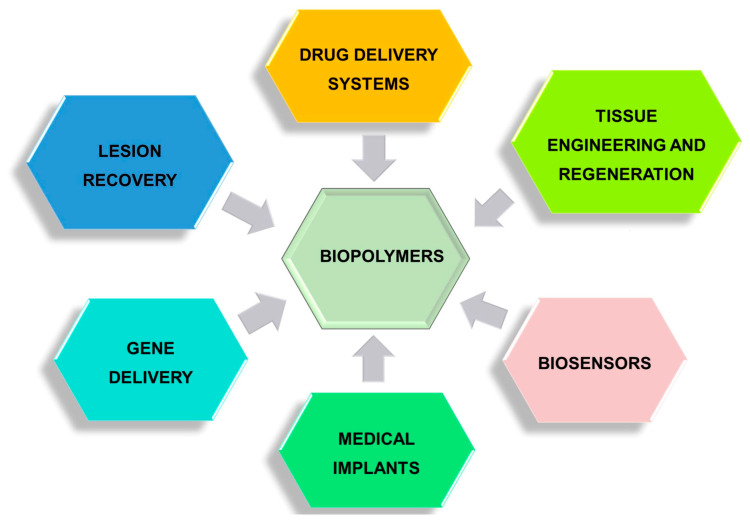
Main biomedical applications of biopolymers.

The applications of biomass polymers are economically, socially, and environmentally sustainable, finding utility across various domains. Ongoing extensive research further explores their potential in diverse fields. Their inherent biocompatibility, biodegradability, and minimal immune response induction position them as promising candidates for applications in TE, as well as in drug and gene delivery systems (Figure 24) [194].

Current advancements in biopolymers have garnered attention across numerous domains due to their improved features and facile commercialization. Typical biopolymers, including elastin, silk, chitosan, keratin, and collagen, have been strategically mixed with synthetic polymers to amplify their actions as composites. The escalating demand for biodegradable natural polymers is particularly notable in the production of packaging film materials, with applications spanning medical, pharmaceutical, and food industries. This contemporary trend emphasizes a burgeoning focus on biopolymers, especially the synergistic blend of synthetic and natural polymers in composite materials. In the medical and pharmaceutical sectors, these polymers play pivotal roles in gene therapy, BTE, and cell-based transplantation, contributing to the development of products such as implantable medical devices, 3D scaffolds, artificial skin, wound dressing materials, and dialysis systems (Table 2) [25,38,195,196].

### 4.1. Drug Delivery Systems

DDSs encompass the conveyance of natural compounds, genes, or synthetic pharmaceutical drugs to the accurate location without inducing negative effects on biological systems. DDSs necessitate comprehensive considerations, including high drug loading, cellular uptake, programmed target specificity, clearance, metabolism, pharmacokinetics, toxicity, and excretion. An ideal system should enhance drug efficiency and enable controlled release from a biocompatible nanocarrier, promoting patient compliance. Passive accumulation at the target site, facilitated by the enhanced permeability and retention (EPR) effect, contributes significantly to DDS efficacy. Traditional DDSs, characterized by immediate release and potential toxicity, often require frequent administration for therapeutic levels. To address these limitations and enhance pharmacokinetics, second- and third-generation DDSs explore modified particle surfaces for improved stealth effects, utilizing hydrophilic blocks like PEG to reduce plasma protein adsorption and rapid clearance. Stealth effects, influenced by factors such as size, shape, and core composition, increase blood circulation and accumulation in highly vascularized areas. Despite these advancements, the clinical translation of formulations based on biodegradable polymers remains limited [231,232].

Biopolymers have become integral in pharmaceutical applications, serving as accurate DDSs with diverse structures for various physiological and medical needs. Structural, protective, and reserve polysaccharides exhibit capability in constructing conjugates with lipids and proteins, facilitating drug transport. Common biopolymers like chitosan, fibroin, starch, gelatin, cellulose, and collagen are harnessed for drug delivery through suspensions, employing methods such as freeze-drying, microemulsion, electrospraying, and supercritical fluid extraction. Biopolymer composites, labeled as excipient materials, are gaining attention in the pharmaceutical industry for drug delivery due to their renewable characteristic, biodegradability, endurance, and reduced toxicity. The focus on targeted drug delivery systems (TDDSs) using polymeric DDSs is increasing, exploring avenues like elastin-like polypeptides (ELPs) for intra-articular delivery and albumin microspheres for controlled drug release. BNC shows promise in delivering proteins with maintained integrity and activity, emphasizing controlled release kinetics, biocompatibility, and hydrophilicity. The abundance of naturally available biopolymers facilitates the cost-effective development of hydrogels and nanogels through various cross-linking polymerization techniques, offering potential applications in cancer treatment [204,205,206,207,208]. Various nanomaterials, encompassing organic polymers and inorganic compounds, have been explored as transportation for DDSs, including liposomes, dendrimers, polymersomes, NPs, nanogels, polymer micelles, nanofibers, nanocapsules, and nanocomposites. These nanocarriers play a crucial role in drug delivery, but precise adjustments in shape, size, porosity, and polydispersity, as well as in surface charge and characteristics, are necessary for their specific applications [233,234,235,236,237,238,239,240,241]. Drug loading and encapsulation efficiencies (DLE and DEE) are crucial parameters in the representation of DDSs. The encapsulation efficiency, drug release profile, and overall performance of polymeric NPs or self-assembled nanoaggregates are influenced by factors like shape, size, surface features, charge, stimuli responsiveness, and polymer biodegradation kinetics. The optimization of these parameters is essential for achieving regulated drug delivery to target sites with optimal doses. Experimental research and theoretical modeling are conducted to configure NPs with specific control over drug discharge, offering expansive and promising applications in clinical medicine. An optimal NP-based delivery system should exhibit an elevated loading capacity, accomplished through drug incorporation during NP preparation or post-incubation diffusion. Understanding drug release mechanisms, influenced by desorption, diffusion, and erosion of the NP matrix, is vital for tailoring drug delivery kinetics. The kinetics of drug delivery are influenced by the biodegradation, diffusion, solubility, and loading effectiveness of the matrix materials. For biodegradable polymers, drug dissolution, swelling, erosion, and diffusion may occur at the same time, inducing zero-order release kinetics, while NP size influences the release pattern [242,243]. The regular course of drug administration via oral ingestion faces challenges with hydrophobic drugs exhibiting reduced bioavailability and protein-based drugs being exposed to enzymatic disintegration in the GI tract. To address this, innovative DDSs are engineered for controlled and targeted release, encapsulating or solubilizing drugs using nanosized elements like inorganic NPs, dendrimers, polymersomes, polymer micelles, liposomes, solid lipid dispersions, and mesoporous materials. Amphiphilic block and graft biomass polymers play a crucial role in these systems, forming nanostructures with enhanced drug-loading ability. Thermo- and pH-responsive polymers are commonly engaged, reacting to the acidic pH of tumor cells and increased concentrations of glutathione (GSH) triphosphate, making them suitable for targeted drug delivery. Biomass polymers, through controlled degradation into biocompatible by-products, offer constant discharge at targeted locations within therapeutic concentration ranges. The provocation lies in designing multiple-functionalized DDSs to ensure on-request, manageable drug dispensation under various external stimuli [196,244,245,246,247,248].

### 4.2. Gene Delivery

Gene therapy is a promising approach to address various metabolic, neoplastic, cardiovascular, neurological, and genetic disorders. It utilizes deoxyribonucleic acid (DNA) and ribonucleic acid (RNA)—therapeutic gene molecules—to modify mutated, absent, or abnormal genes. To overcome challenges like the brittle characteristic of therapeutic genes and biological impediments, non-viral vectors, including lipid-based nano-assemblies and cationic polyelectrolytes, are developed for gene delivery. Polyplexes formed by cationic polyelectrolytes like polyethylene imines and poly(L-lysine) show potential, but a careful consideration of polymer design, degradability, and toxicity is crucial. Non-toxic protein-based vectors, such as albumin and gelatin, are widely used due to their biocompatibility and biodegradability. Cationic polysaccharides, such as chitosan, though limited by solubility, are explored, with chemical alterations to enhance gene complexation [209,210,211].

### 4.3. Lesion Recovery

Wound healing is an intricate biological and cellular mechanism, including phases of inflammation, hemostasis, proliferation, and remodeling triggered by tissue severance. Cutaneous injuries create vulnerabilities for pathogenic bacteria, leading to virulence factor production that hinders tissue integrity, often associated with biofilm formation. Various dressings are employed for severe wounds, possessing high absorption capacity, wound visibility, pain-free removal, and non-allergenic properties. Biomass polymers are utilized in wound dressing formulations such as hydrogels, films, hydrocolloids, membranes, and foams. Polysaccharides like HA, cellulose, chitosan, and alginate stand out as adaptable biomacromolecules due to their increased chelation ability, non-toxicity, biodegradability, biocompatibility, multifunctional groups, and simple chemical alteration, making them effective in the management of cutaneous infections [212,213,214,215,216,217,218,219,220].

### 4.4. Targeted Diagnosis

Targeted therapy involves the use of ligand-functionalized NPs to accurately recognize receptors overexpressed in malignant tumor cells, enabling tumor-selective DDSs. Various ligands, including antibodies, aptamers, transferrin, peptides, and folic acid, have been explored to enhance the specificity of DDSs. NPs derived from biomass polymers, particularly chitosan, show promise in anti-tumor targeting due to their ability to promote cellular uptake and adhesion to mucosal surfaces. This targeted approach aims to improve drug release directly to cancer cells, enhancing therapeutic efficacy [221,222,223,224,225,226,227].

### 4.5. Tissue Engineering and Regeneration

TE integrates regulations of engineering and medical sciences to elaborate biological tissue replacements for improving, maintaining, or restoring function. The engineered tissue can be developed in vivo or in vitro and transplanted, serving diagnostic purposes as well. Scaffolds, valuable in this particular field, rely on biodegradable polymers, such as biomass polymers, from natural sources. Perfectly, scaffolds should be mechanically powerful, biocompatible, biodegradable without toxic byproducts, possess an appropriate surface morphology for cell interaction, and sustain cell attachment, proliferation, and differentiation. Proteins like silk, gelatin, and collagen, along with polysaccharides like chitosan, HA, and alginate, are universal natural scaffold materials. Nevertheless, due to the intricate structure of biomass polymers and concerns like immunogenicity, synthetic polymers like polyurethanes, polyphosphazenes, polyanhydrides, and polyesters gain significance. In BTE, the challenge lies in regenerating bone deficiencies caused by tumors, fractures, or trauma, where polymers, ceramic materials, and metals serve as scaffolds to encourage new tissue formation. Inorganic materials like titanium and steel and degradable biomass polymers show promise, with PHA composites, acrylate polymer polyblends, and magnesium-based compounds demonstrating excellent mechanical and cell adhesion features for orthopedic applications [228,229,230,231,232,233].

### 4.6. Biosensors

Advanced diagnostic instruments often rely on automated analyzers, but their maintenance is both expensive and time-consuming. The demand for more accelerated, compact, and cost-effective devices in laboratory testing has led to the rise in biosensors. These diagnostic tools recognize precise biochemicals by utilizing immobilized biomolecules, such as receptors, antibodies, or enzymes, on electrodes, offering elevated specificity, maneuverability, utilizer accessibility, and rapid response duration. Biosensors, incorporating biological constituents and transducers, can monitor medical conditions through the examination of clinical samples or real-time physiological modifications inside the human organism. Chitosan-based films have been instrumental in enhancing the sensitivity of biosensors, particularly in applications like cholesterol detection [234,235,236,237,238].

### 4.7. Medical Implants

Various biopolymers, like chitosan and PLA, have found extensive use in pharmaceutical purposes, due to their biocompatibility and biodegradability when employed as implantable medical devices. For example, chitosan serves as implants in surgery (e.g., nerve regeneration), cardiology (e.g., heart valves), and ophthalmology (e.g., contact lenses). Composites of chitosan are applied for tissue regeneration, bioartificial livers, and bone scaffolds, whereas HA implants aid in tissue maturation in otolaryngology. Gelatin serves multiple purposes, such as 3D biomatrices in dermatology, bone replacement in orthopedics, and grafts in cardiology. Collagen, a ubiquitous biopolymer in mammals, is utilized as cardiovascular implants, bone marrow, and bone scaffolds, while PHAs are employed in nerve, vascular, and esophageal implants. Moreover, polyhydroxybutyrate (PHB) serves as cell culture scaffolds and surgical implants. Biopolymers derived from biomass are designed via techniques such as leaching, freeze-drying, 3D bioprinting, electrospinning, and casting, serving as medical implants, such as barrier membranes and stents, as well as carriers in cell, gene, drug, and growth factor delivery systems [203,239,240,241].

### 4.8. Challenges

Using biomass polymers in pharmaceutical applications has several challenges that researchers must overcome. Although the vast majority of them possess desirable properties such as biocompatibility and biodegradability, their use comes with a set of limitations. Proteins (collagen, gelatin, albumin) can potentially trigger immune responses in the body, leading to allergic reactions or even rejection in the case of their use as carrier systems [249,250]. There are some good strategies to minimize immunogenic responses, such as purification techniques or surface modification, but also in this case, it must be considered that traditional purification methods may affect protein properties and integrity [251,252]. The same challenge of maintaining structural integrity is encountered when applying the processes of sterilization techniques (heat, irradiation) [253]. An alternative sterilization that preserves protein functionality while eliminating microbial contaminants for maintaining product quality and safety was developed [254,255]. Other challenges in the case of protein biopolymers were the ethical or cultural objections that involve the use of animal-derived proteins in pharmaceutical products, particularly those sourced from pigs or cows [256]. Marine or plant-based sources are good alternatives to solve consumer preferences and address ethical concerns [256].

Polymer stability [257] is another important parameter in pharmaceutical applications. Maintaining structure during processing and formulation can be challenging. For example, collagen may undergo denaturation or degradation, affecting its stability and performance as a drug carrier. Stabilization techniques, such as cross-linking or encapsulation within protective matrices such as those discussed above, are frequently applied [258].

The cost of sourcing high-quality biopolymers and any additional processing steps can influence the overall affordability of biopolymer-based pharmaceuticals. For example, lignin extraction methods, such as those from paper and pulp industries or lignocellulose biorefineries, may not be optimized for pharmaceutical-grade lignin production [259,260]. Developing efficient and sustainable extraction processes, as well as exploring alternative lignin sources, can decrease the overall price. Therefore, researchers must improve the characteristics of biopolymers to increase their functionalities and pharmaceutical applicability.

## 5. Conclusions and Perspectives

The investigation into the domain of biomass polymers for pharmaceutical applications reveals an exciting pathway toward the creation of innovative, sustainable healthcare technologies. The sophisticated chemical modifications applied to these biopolymers—such as cross-linking, functionalization, and conjugation, along with the engineering of complex structures like IPNs, grafts, and block copolymers, and PICs—mark a significant step forward in enhancing their versatility and compatibility with biological systems. This progress lays the groundwork for their widespread application in a variety of pharmaceutical and biomedical contexts, including drug and gene delivery, lesion healing, precision diagnosis, TE, biosensing, and the development of medical implants. This exploration underscores the transformative potential of biomass polymers in medicine and pharmaceutical science, offering novel approaches to address enduring challenges in these fields.

Looking forward, several pivotal areas warrant further investigation to fully harness the capabilities of biomass polymers. The development of advanced DDSs that offer controlled release and biodegradation kinetics tailored to physiological conditions is of paramount importance. Optimizing gene delivery vectors to achieve high efficiency with minimal cytotoxicity, expanding research on the integration of biomass polymers with living tissues for regeneration and TE, and advancing biosensor technologies for the sensitive detection of disease biomarkers represent critical frontiers in this domain. Furthermore, the evaluation of biomass-polymer-based medical implants in vivo to assess their long-term biocompatibility and functionality will be crucial for their clinical application.

Moreover, tackling the challenges of sustainable biomass sourcing and the scalable production of these polymers will ensure their accessibility and economic feasibility. Encouraging interdisciplinary collaboration will also be instrumental in pioneering new biomass polymer formulations tailored for specific medical applications. Through dedicated research and collaborative innovation, the promising future of biomass polymers in enhancing pharmaceutical and biomedical solutions can be fully realized, steering us toward a future where these sustainable and effective technologies become a cornerstone of healthcare advancements.

## Figures and Tables

**Table 1:** Examples of enhanced biopolymer properties for pharmaceutical applications through chemical modifications.

Biopolymers		Chemical Modifications	Enhanced Properties
Polysaccharides	Chitin and chitosan	IPNs [169], grafting [170], cross-linking quaternization [171], hydroxypropylation carboxymethylation, sulfation, esterification	Improved mechanical strength and stability; prolonged release time; enhanced interaction with other molecules, water absorption capacity, and resistance to enzymatic degradation; and increased surface activity
	Cellulose		
	Hyaluronic acid		
	Alginate		
	Pectins		
Proteins	Collagen	Glutaraldehyde cross-linking, carbodiimide cross-linking, glycosylation, hydroxylation, PEGylation [172], acetylation	Increased stability and resistance to enzymatic degradation, improved mechanical properties, mimics the native extracellular matrix, enhanced solubility
	Gelatin	PEGylation, hydroxylation, glycosylation, acetylation [173], cross-linking (glutaraldehyde [113] or transglutaminase [174])	Resistance to enzymatic degradation; increased bioactivity; and enhanced solubility, stability, and mechanical properties
	Albumin	Site-specific PEGylation [175], drug conjugation [176]	Minimized interference with albumin’s binding and transport functions, enhanced drug pharmacokinetic properties, improved biodistribution, and reduced toxicity

IPNs: interpenetrating polymer networks; PEG: polyethylene glycol.

**Table 2:** Examples of biopolymers and their pharmaceutical/biomedical applications.

Biopolymers	Applications	References
Chitosan Fibroin Starch Gelatin Cellulose Bacterial nanocellulose Collagen Biopolymer composites Elastin-like polypeptides Albumin microspheres	Drug delivery systems	[197,198,199,200,201,202,203,204,205,206,207,208,209]
Polyethylene imine Poly(L-lysine) Albumin Gelatin Chitosan	Gene delivery	[202,210,211]
Hyaluronic acid Cellulose Chitosan Alginate	Lesion recovery	[212,213,214,215,216,217,218,219,220]
Chitosan nanoparticles	Targeted diagnosis	[221,222,223,224,225,226,227]
Silk Gelatin Collagen Chitosan Hyaluronic acid Alginate Polyurethanes Polyphosphazenes Polyanhydrides Polyesters Polyhydroxyalkanoates Acrylate polymers polyblends	Tissue engineering and regeneration	[228,229,230,231,232,233]
Chitosan-based films	Biosensors	[234,235,236,237,238]
Chitosan Polylactic acid Gelatin Collagen Polyhydroxyalkanoates Polyhydroxybutyrate	Medical implants	[203,239,240,241]

## Data Availability

Data described in the manuscript will be made publicly and freely available without restriction at https://docs.google.com/document/d/1Lz89uNfXp_TYbX6vJFAr0_JiolruP1Tk/edit?usp=sharing&ouid=106104952021876684289&rtpof=true&sd=true (accessed on 5 March 2024).
